# Machine learning-based predictive models and drug prediction for schizophrenia in multiple programmed cell death patterns

**DOI:** 10.3389/fnmol.2023.1123708

**Published:** 2023-03-13

**Authors:** Yu Feng, Jing Shen

**Affiliations:** ^1^The University of New South Wales, Kensington, NSW, Australia; ^2^The University of Melbourne, Parkville, VIC, Australia; ^3^The Affiliated Jiangsu Shengze Hospital of Nanjing Medical University, Nanjing, China

**Keywords:** schizophrenia, machine learning, diagnostic modeling, drug prediction, programmed cell death, apoptosis, ferroptosis, autophagy

## Abstract

**Background:**

Schizophrenia (SC) is one of the most common mental illnesses. However, the underlying genes that cause it and its effective treatments are unknown. Programmed cell death (PCD) is associated with many immune diseases and plays an important role in schizophrenia, which may be a diagnostic indicator of the disease.

**Methods:**

Two groups as training and validation groups were chosen for schizophrenia datasets from the Gene Expression Omnibus Database (GEO). Furthermore, the PCD-related genes of the 12 patterns were extracted from databases such as KEGG. Limma analysis was performed for differentially expressed genes (DEG) identification and functional enrichment analysis. Machine learning was employed to identify minimum absolute contractions and select operator (LASSO) regression to determine candidate immune-related center genes, construct protein–protein interaction networks (PPI), establish artificial neural networks (ANN), and validate with consensus clustering (CC) analysis, then Receiver operating characteristic curve (ROC curve) was drawn for diagnosis of schizophrenia. Immune cell infiltration was developed to investigate immune cell dysregulation in schizophrenia, and finally, related drugs with candidate genes were collected *via* the Network analyst online platform.

**Results:**

In schizophrenia, 263 genes were crossed between DEG and PCD-related genes, and machine learning was used to select 42 candidate genes. Ten genes with the most significant differences were selected to establish a diagnostic prediction model by differential expression profiling. It was validated using artificial neural networks (ANN) and consensus clustering (CC), while ROC curves were plotted to assess diagnostic value. According to the findings, the predictive model had a high diagnostic value. Immune infiltration analysis revealed significant differences in Cytotoxic and NK cells in schizophrenia patients. Six candidate gene-related drugs were collected from the Network analyst online platform.

**Conclusion:**

Our study systematically discovered 10 candidate hub genes (*DPF2*, *ATG7*, *GSK3A*, *TFDP2*, *ACVR1*, *CX3CR1*, *AP4M1*, *DEPDC5*, *NR4A2*, and *IKBKB*). A good diagnostic prediction model was obtained through comprehensive analysis in the training (AUC 0.91, CI 0.95–0.86) and validation group (AUC 0.94, CI 1.00–0.85). Furthermore, drugs that may be useful in the treatment of schizophrenia have been obtained (Valproic Acid, Epigallocatechin gallate).

## 1. Introduction

Schizophrenia is a chronic psychological disorder identified by hallucinations, delusions, and confusion, as well as motivational and cognitive dysfunction (Kahn et al., 2015). Schizophrenic patients face a fatality risk about two to three times greater than the standardized rate of mortality, and this difference increases every year (McGrath et al., 2008). Suicide was considered one of the major death causes in patients with schizophrenia over the past five-years World Health Organization study of psychiatric patients (White et al., 2009), and attempted suicide rates in patients with schizophrenia were 10–20 times higher (Balhara and Verma, 2012). A large number of studies have demonstrated that environmental factors play an influential role in the pathogenesis of schizophrenia. The findings of these studies suggest that the disorder may be caused by multiple factors, including intrauterine infections, micronutrient deficiencies, and fetal hypoxia. As a result, these factors can interact in complex ways with the macro-structural environment, including psychological, social, cultural, and economic contexts, in order to increase the risk of schizophrenia (Brown, 2011).

Accidental cell death (ACD) and programmed cell death (PCD) are the two divisions of cell death. PCD is characterized by distinct morphological features and competency-dependent biochemical mechanisms and is considered an important component of a variety of processes (Elmore, 2007). Twelve types have now been identified in studies of PCD, including Apoptosis, Pyroptosis, Ferroptosis, Autophagy, Necroptosis, and Cuproptosis Parthanatos Entotic cell death, Netotic cell death, Lysosome-dependent cell death, Alkaliptosis and Oxiptosis (Zou et al., 2022). The introduction to Gasdermin family (Yu et al., 2021) and the connection of innate immunity and disease with pyroptosis have increased the scope of its research. The accumulation of reactive lipid-based oxygen species resulting in a regulatory form of cell death through an iron-dependent process was termed as the iron death in 2012 (Hirschhorn and Stockwell, 2019). Similarly, the accumulation of Cu in mitochondria causing the aggregation of lipidated TCA cycle enzymes *via* direct Cu binding led to copper death which is the most recent form of cell death (Cobine and Brady, 2022).

PCD causes abnormal neuronal numbers and pathological neurodevelopment not only in typical neurodegenerative diseases such as Alzheimer’s disease but also in many neurodevelopmental disorders such as schizophrenia and autism (Margolis et al., 1994). However, there have been few detailed functional studies on PCD in schizophrenia, therefore this research dealt with the development of a predictive model with good diagnostic efficacy using PCD-related genes, as well as hypotheses about the possibility of other pharmacological treatments.

## 2. Materials and methods

### 2.1. Materials

**Figure 1:**
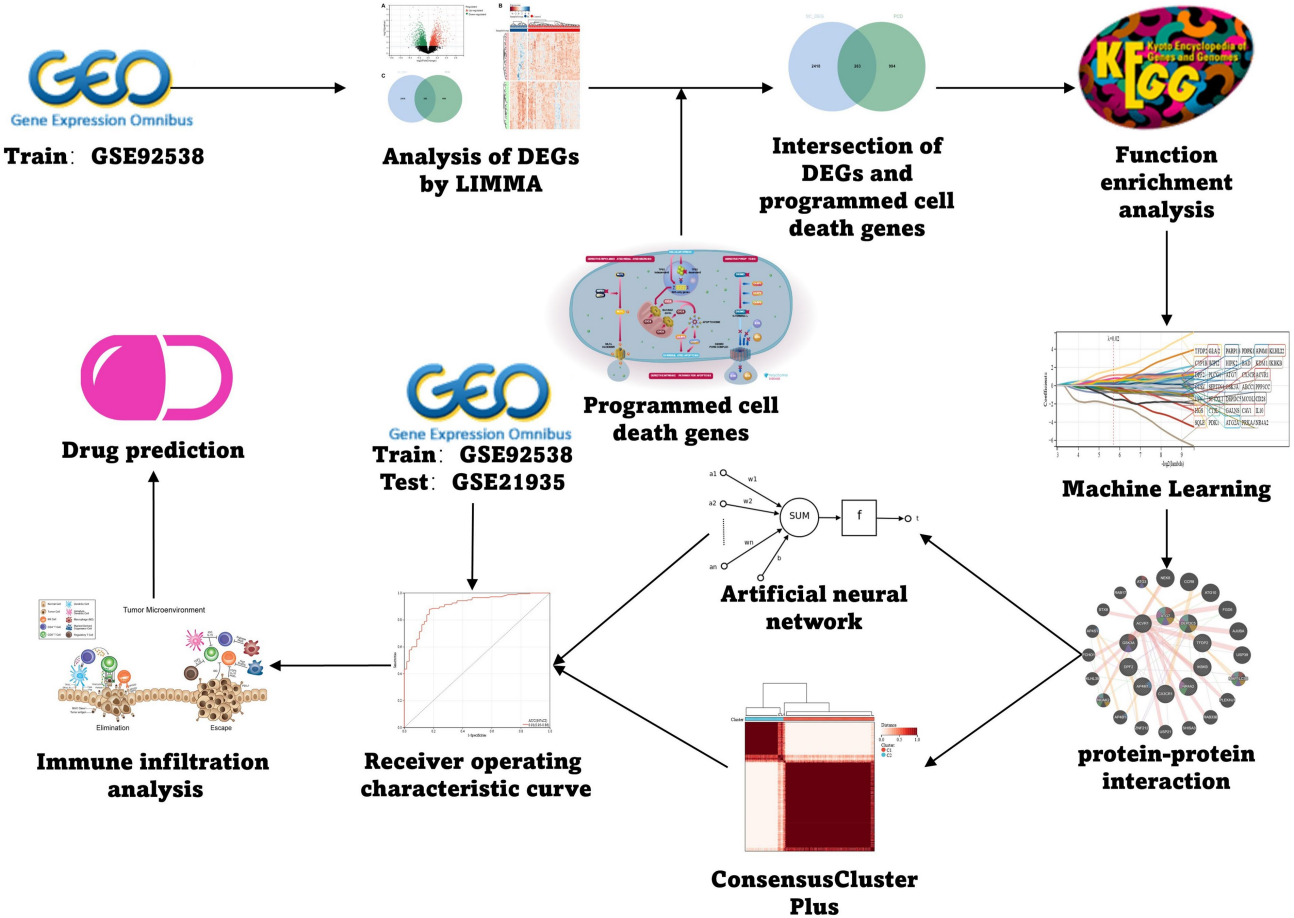
Flow chart.

The schizophrenia datasets utilized as training and test group were the GSE92538 and the GSE21935, respectively, which were retrieved from the GEO database^1^ (Barrett et al., 2012). Genes linked to PCD were collected from the GSEA gene set, KEGG, and relevant literature to finally obtain 1,257 related genes, which were collated according to different types (Appendix Table 1), and the specific flow was illustrated (Figure 1).

### 2.2. Screening for differentially expressed genes

Limma (linear models for microarray data) (Sokhansanj et al., 2004) is a generalized linear model-based method for screening differential expression. The genes with differential expression between the comparison and control groups were obtained by employing the package Limma of the R software v3.40.6 for differential analysis. In this study, |log2 fold change (FC)| > 1 and *p* < 0.05 were selected as criteria for identifying differentially expressed genes (DGE) by Limma package, and heat maps and volcano maps of DEG in schizophrenia were visualized by sangerBox, respectively (Shen et al., 2022).

### 2.3. Gene function enrichment analysis

The genes linked with schizophrenia and PCD were determined in order to analyze gene function enrichment by utilizing the Venn diagram to cross-screen the DEG and PCD-related genes of schizophrenia that had been determined by the above treatment. In order to perform gene, set functional analysis the KEGG rest API^2^ was utilized and the gene annotation of the most recent KEGG pathway was retrieved. The R software package org.Hs.eg.db v3.1.0 was utilized for the genes’ GO annotations (Carlson, 2022) which were utilized for background mapping and cluster-profiler R software package v3.14.3 (Yu et al., 2012) was employed to perform enrichment analysis for obtaining the results of the gene set enrichment. Based on gene expression profiles and phenotypic groupings, the lowest gene range was 5 and the highest was set at 5000, with a *p* value of <0.05 and FDR of <0.1 considered statistically significant.

### 2.4. Machine learning screening for schizophrenia and PCD-related candidate genes

Machine learning algorithms were adopted to further filter candidate genes for SC diagnosis. LASSO is a regression method for selecting a variable to improve the predictive accuracy and is also a regression technique for variable selection and regularization to improve the predictive accuracy and comprehensibility of a statistical model (Yang et al., 2018). LASSO-COX regression was analyzed by integrating the data of survival time, survival status, and gene expression data utilizing the Glmnet R package (Zhang et al., 2019) analysis. Moreover, 10-fold cross-validation was executed to establish the best model. Differential expression profiling was used to examine candidate genes, and 10 genes with the most significant differences were chosen to build a diagnostic model.

### 2.5. Construction of protein–protein interaction networks (PPI)

The PPI was constructed utilizing a convenient GeneMANIA^3^ website which is utilized to generate gene function hypotheses, gene lists analysis, and determination of gene priorities for performing functional analysis (Franz et al., 2018).

### 2.6. Diagnostic model validation

ROC analysis using pROC (Robin et al., 2011) in the R package was performed to obtain AUC. SangerBox was used for visualizing the final AUC results which were obtained by employing the CI function of pROC to assess the confidence intervals and AUC values. The signature genes were observed for expression in the training (GSE92538) and test groups (GSE21935). Furthermore, a neuralnet (Beck, 2018) in the R software was utilized to construct an artificial neural network for the characteristic genes acquired by the methods mentioned above to build a diagnostic model of high precision. Additionally, the “ConsensusClusterPlus” package (Wilkerson and Hayes, 2010), which made use of agglomerative km clustering with 1-Pearson correlation distances and repeated sampling of 80% of the data 10times, was employed to observe the prediction effect using empirical cumulative distribution function plots.

### 2.7. Immuno-infiltration analysis

A method based upon the gene set signature, the ImmuCellAI,−was utilized for the precise estimation of the abundance of 24 types of immune cells which included 18 subsets of T-cells, from data on gene expression (Miao et al., 2020). Immuno-infiltration analysis was performed *via* the online website ImmuCellAI^4^ and correlation was calculated using the spearman coefficient (Pripp, 2018). The comparison regarding the proportion of diverse types of immune cells between SC and control groups was visualized *via* the box plot.

### 2.8. Drug prediction

Gene-drug interaction networks were created using the Network analyst^5^ (Zhou et al., 2019).

## 3. Results

### 3.1. Screening of differentially expressed genes in schizophrenia

**Figure 2:**
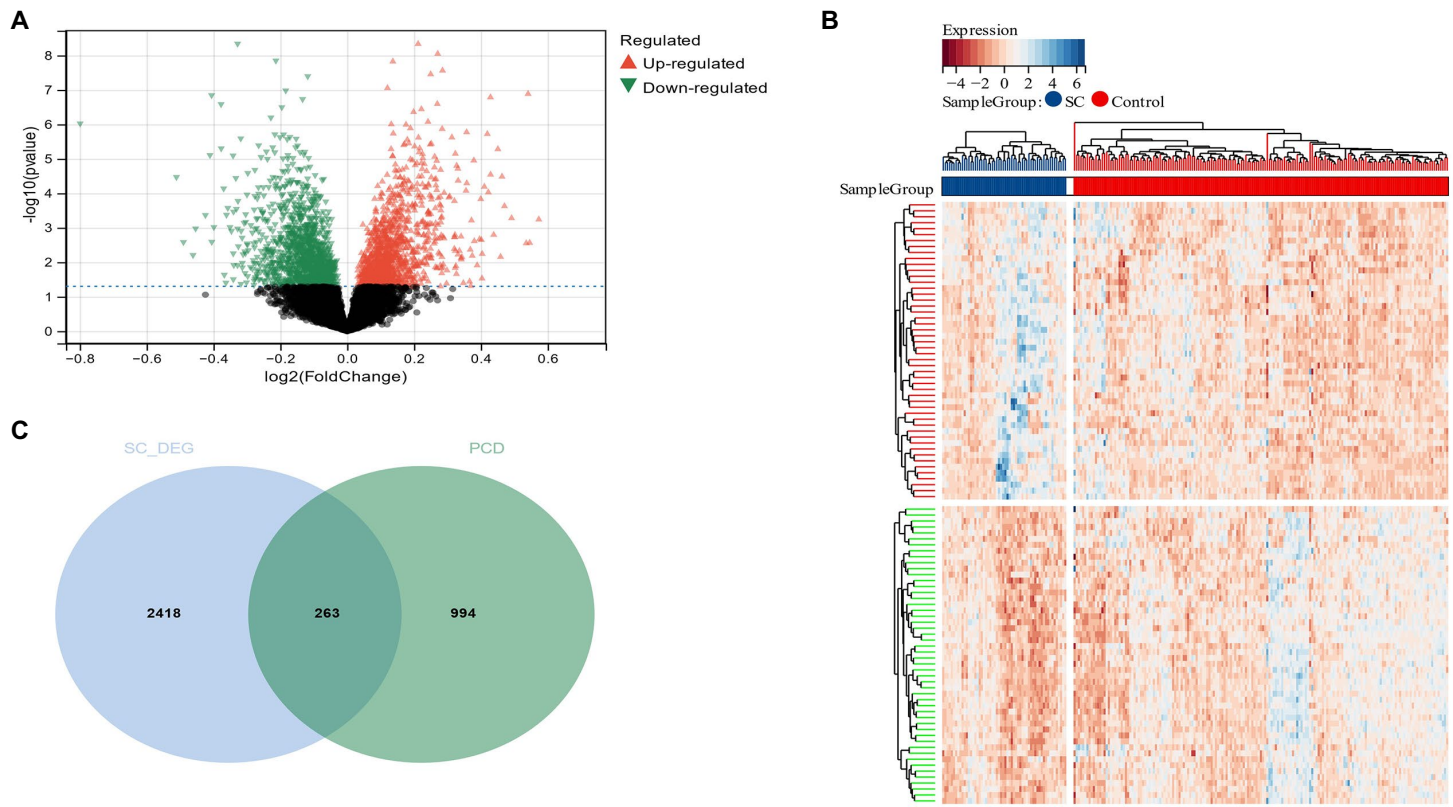
**(A)** The volcano plot shows all DEGs of schizophrenia, of which red and green triangles refer to significant DEGs; **(B)** Based on the SC dataset, the heatmap displays the top 50 DEGs that have been upregulated or downregulated. Rows represent intersections of genes, while columns represent SC cases or controls. The blue and red colors represent genes whose expression has been upregulated or downregulated; **(C)** Cross-screening of schizophrenia DEG and PCD-related genes yielded relevant candidate genes.

Using the Limma method, a schizophrenia dataset (GSE92538) was identified as enlisting about 2,684 DEG, of which 1,299 were up-regulated and 1,382 down-regulated (Figures 2A,B). Two hundred sixty-three candidate genes associated with schizophrenia and PCD were cross-screened *via* a Venn diagram (Figure 2C).

### 3.2. Functional enrichment analysis of candidate genes associated with PCD in schizophrenia

**Figure 3:**
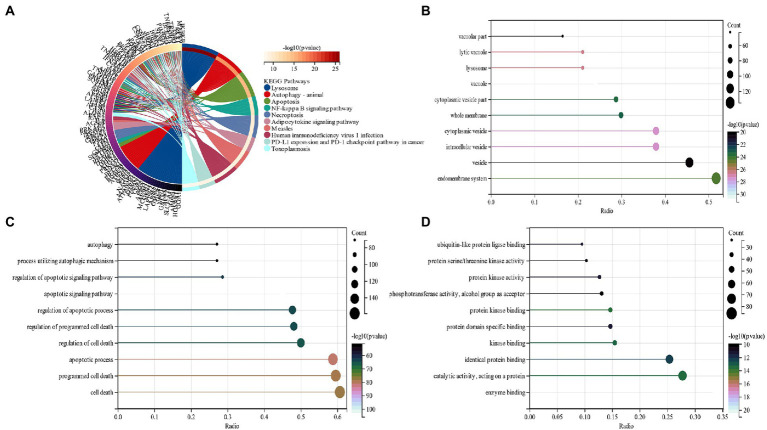
**(A)** An analysis of KEGG pathways at the intersection of genes. Genes enriched in different pathways are represented by different colors; **(B)** GO analysis of cell components of candidate genes (CC); **(C)** GO analysis of the biological process of candidate genes (BP); **(D)** GO analysis of the molecular function of candidate genes (MF).

The functional enrichment analysis of candidate genes was performed, and KEGG analysis displayed that “lysosome,” “Autophagy” and “Necroptosis” pathways depicted predominant enrichment of candidate genes (Figure 3A). In terms of cellular components (CC), GO analysis revealed that the major allocation of candidate genes was in “vacuole,” “cytoplasmic vesicle,” and “intracellular vesicle” (Figure 3B). Major biological processes (BP) of candidate genes include the “apoptotic signaling pathway” and “apoptotic process” (Figure 3C). Molecular function (MF) revealed that candidate genes functioned predominantly in “enzyme binding” and “protein kinase binding” (Figure 3D).

### 3.3. Screening of candidate genes associated with PCD and construction of PPI network in schizophrenia utilizing machine learning

**Figure 4:**
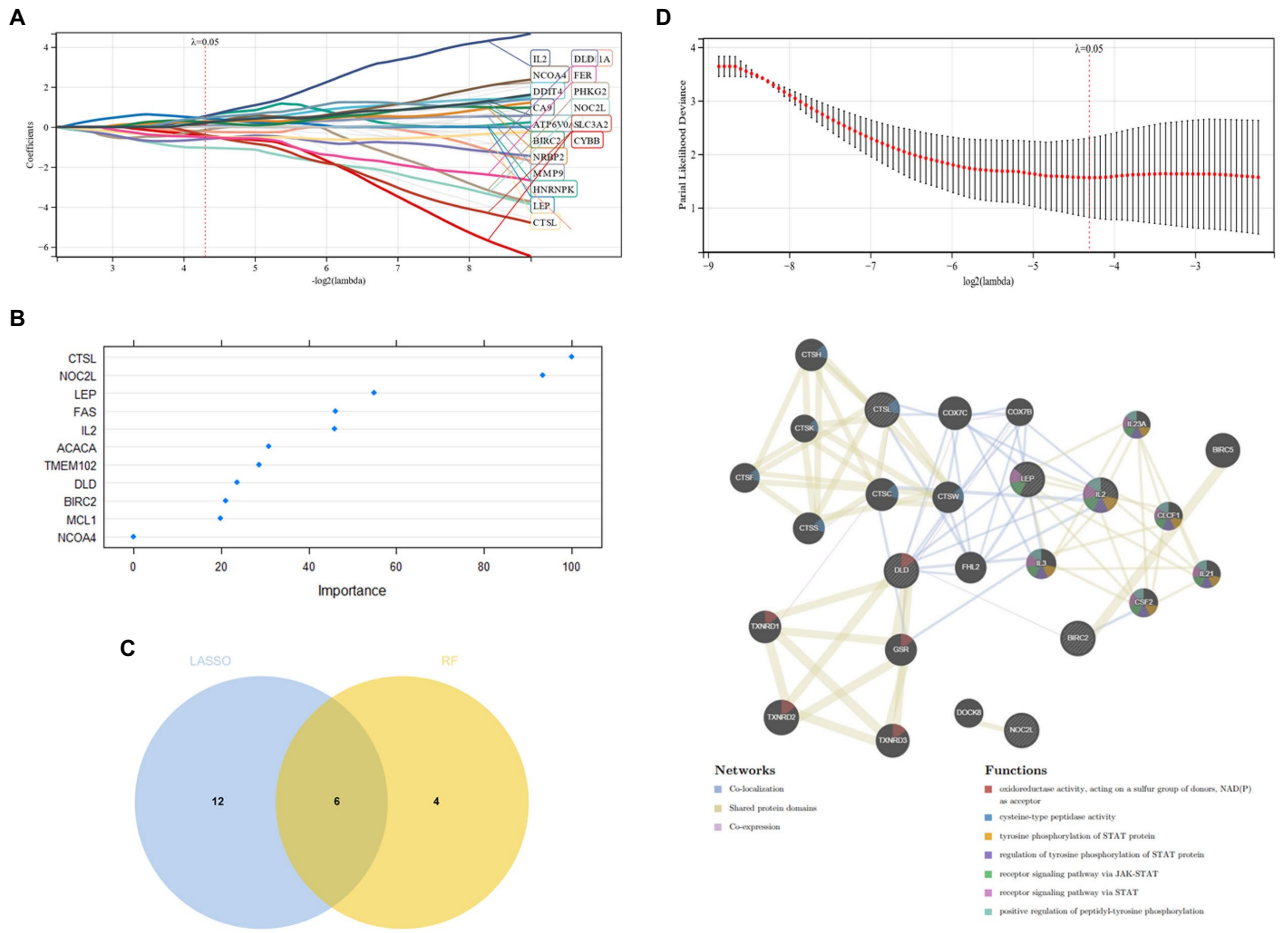
**(A,B)** Candidate gene screening *via* LASSO regression; **(C)** Differential expression profiling of candidate genes; **(D)** PPI network construction of candidate genes.

Candidate genes were screened by the LASSO regression method. Forty-two potential candidate genes were identified from the results (Figures 4A,B). The expression profile analysis of 42 candidate genes was organized to identify the 10most differentially expressed genes for further investigation (*DPF2*, *ATG7*, *GSK3A*, *TFDP2*, *ACVR1*, *CX3CR1*, *AP4M1*, *DEPDC5*, *NR4A2*, *IKBKB*) (Figure 4C; Appendix Table 2), which included six Apoptosis, one Ferroptosis, three Autophagy, one Entotic cell death, one Lysosome-dependent cell death, and one Alkaliptosis. The PPI network was established by these 10 candidate genes, in which Co-expression accounted for 61.7% and Physical Interactions accounted for 31.77%. These genes are mainly involved in mitochondrion disassembly, AP-type membrane coat adaptor complex, cellular response to starvation, organelle disassembly and cellular response to external stimulus (Figure 4D).

### 3.4. Validation of diagnostic model

**Figure 5:**
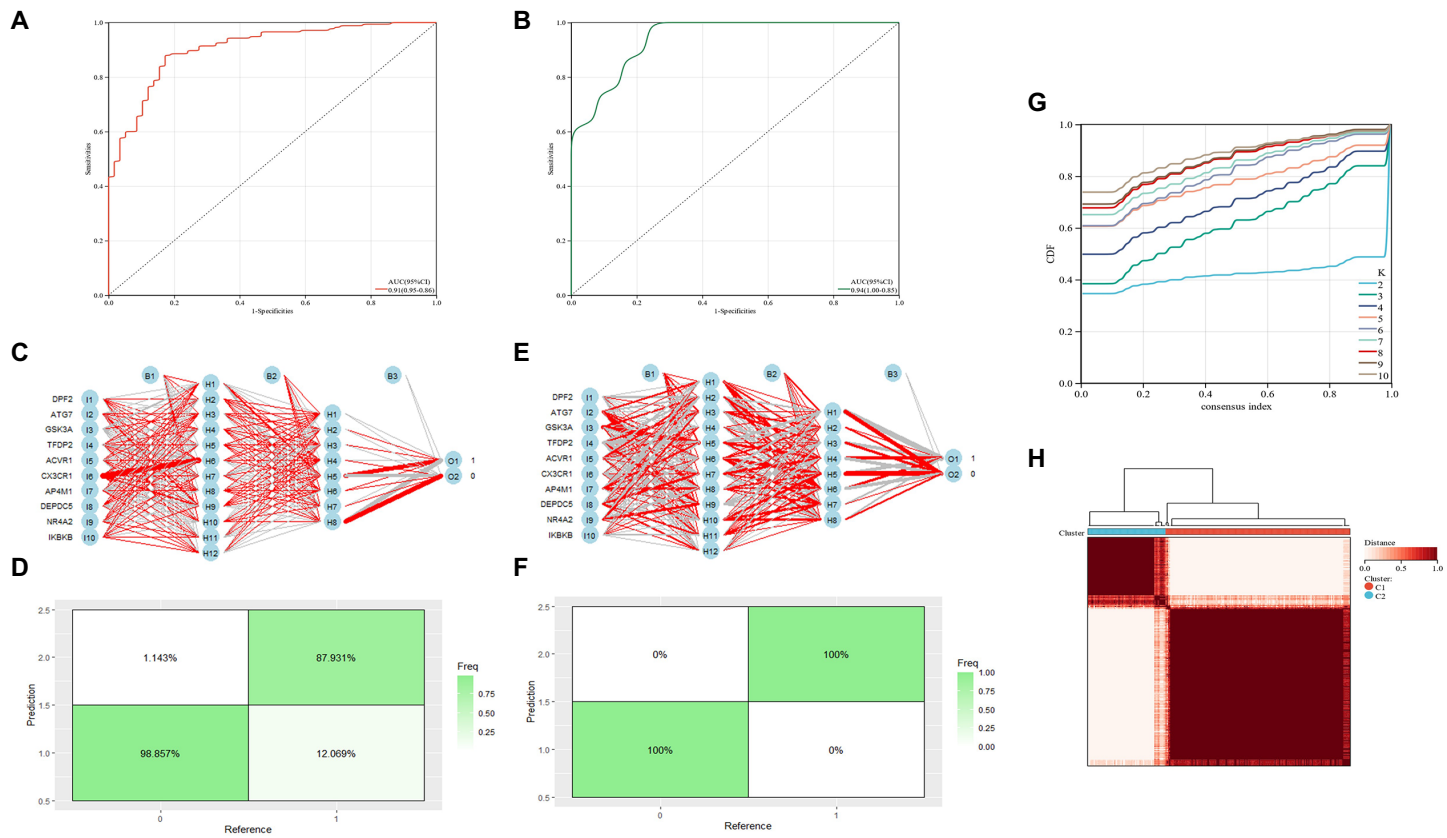
**(A)** Training group ROC curve; **(B)** Test group ROC curve; **(C,D)** Validation of the artificial neural network of the training group; **(E,F)** validation of the artificial neural network of the validation group; **(G,H)** CC analysis of the related gene model.

The diagnostic value of these two candidate genes was validated using ROC curves when all candidates were combined (AUC 0.91, CI 0.95–0.86; Figure 5A). The diagnostic model was placed in the validation group (GSE21935) for validation, and the results showed that it had a very good diagnostic significance (AUC 0.94, CI 1.00–0.85; Figure 5B). Neural networks were constructed by employing candidate genes, and the findings showed that schizophrenia samples could be visibly distinguished from controls by these 10 genes, with an accuracy of 87.931% in the training (Figures 5C,D) and 100% in the validation group (Figures 5E,F). The consensus clustering (CC) analysis of 10 PCD-related gene models was carried out, and differences between different groups were most pronounced when *K* = 2, indicating that schizophrenia samples could be well distinguished from control samples (Figures 5G,H).

### 3.5. Immune cell infiltration analysis

**Figure 6:**
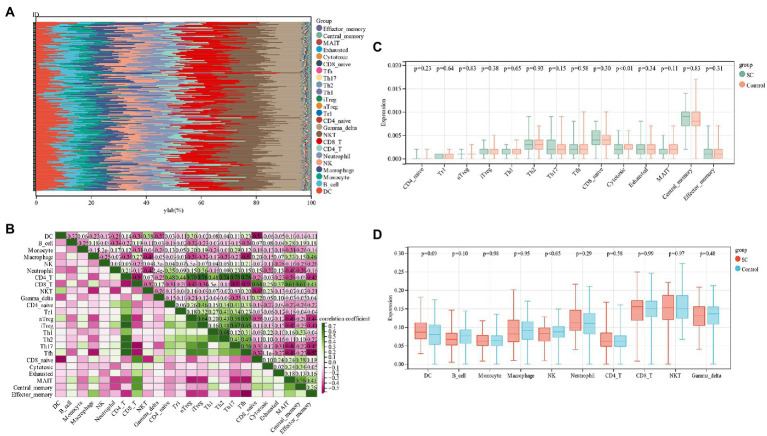
**(A)** Relative percentage of 24 immune cells per sample; **(B)** Correlation between 24 immune cells; **(C,D)** Differences in immune infiltration between schizophrenia samples and control samples.

By functional enrichment analysis we observed that PCD genes could regulate SC pathogenesis and were mainly enriched in immune regulation. These genes could be used as a potential SC diagnostic biomarker by ROC evaluation. In order to better understand how SC is regulated by the immune system, an analysis of immune cell infiltration was performed. The proportion of 24 immune cells in schizophrenia and control samples of the training group (GSE92538) was estimated *via* the ImmuCellAI algorithm (Figures 6A,B). In boxplots, immune cell infiltration was compared between schizophrenia and control samples (Figures 6C,D), and there were significant differences in Cytotoxic and NK cells among schizophrenia patients (*p* < 0.05), low levels of NK cells are found in SC patients.

### 3.6. Drug prediction

**Figure 7:**
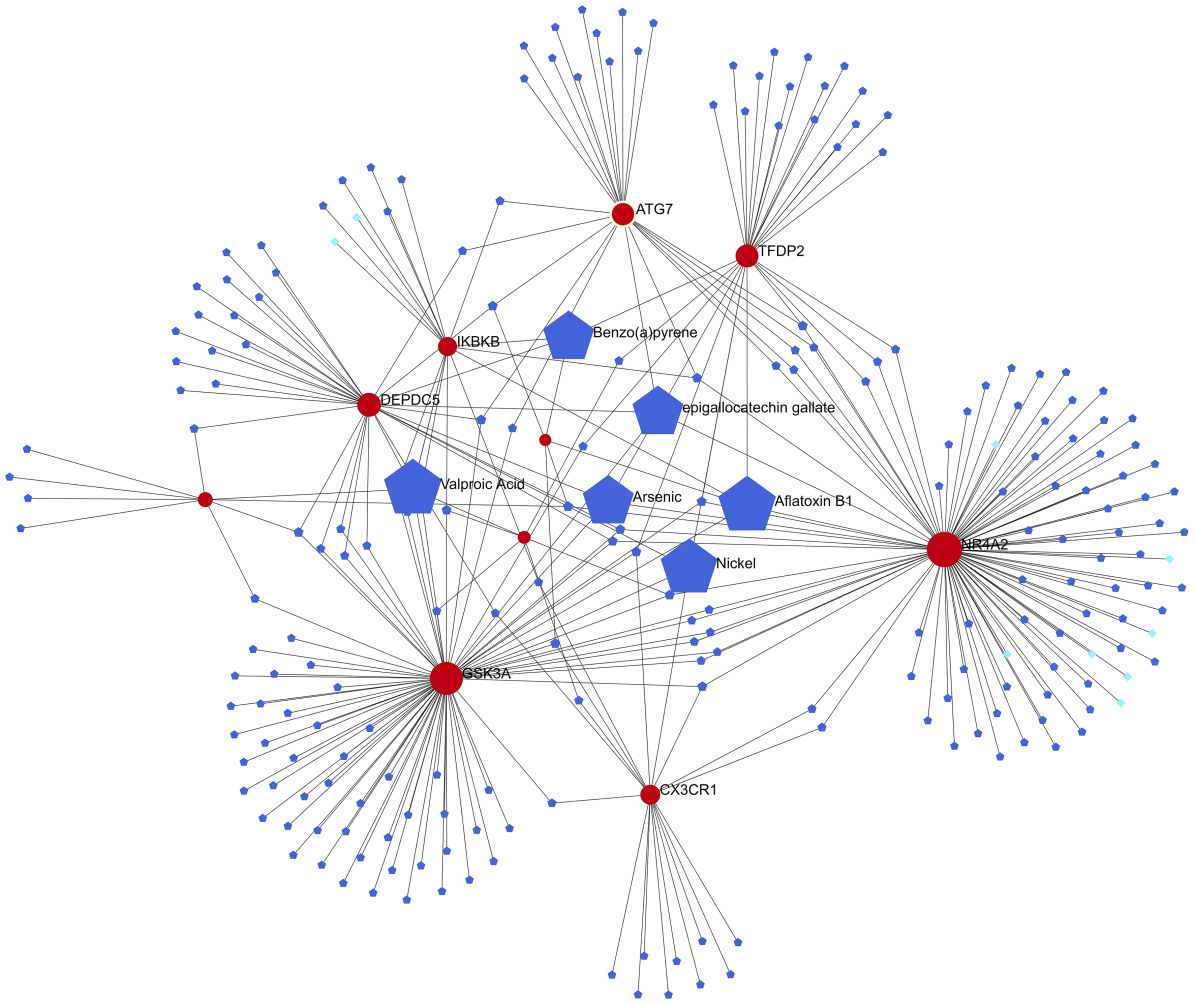
Gene-drug interaction network (red represents candidate genes, light blue data from DrugBank, and dark blue data from Comparative Toxicogenomics Database).

Six of the most relevant agents (Aflatoxin B1, Valproic Acid, Arsenic, Benzo(a)pyrene, epigallocatechin gallate, Nickel) were selected using the Network analyst online platform to construct a gene-drug interaction network based on DrugBank (Wishart et al., 2018) and Comparative Toxicogenomics Database (Davis et al., 2022; Figure 7).

## 4. Discussion

Increasing research suggests a close link between schizophrenia and cell death (Glantz et al., 2006; Yang and Xu, 2020), and the initial thorough examination of 12 different PCD modes in schizophrenia was achieved in this research. It was shown from the results that schizophrenia is most closely linked to Apoptosis, Ferroptosis, Autophagy, Entotic cell death, cell death associated with lysosomes, and Alkaliptosis (Jarskog, 2006; Sragovich et al., 2017; Feng et al., 2022). We evaluated the diagnostic value of PCD in SC patients by integrating bioinformatics analysis and machine learning methods. One of the most noteworthy discoveries is the identification of 10 pivotal candidate genes (DPF2, ATG7, GSK3A, TFDP2, ACVR1, CX3CR1, AP4M1, DEPDC5, NR4A2, and IKBKB).

ATG7 is a Protein Coding gene associated with Ferroptosis, Autophagy, and Entotic Cell Death. Spinocerebellar Ataxia, Autosomal Recessive 31, Fatty Liver Disease andNon-alcoholic 1 are the diseases caused by ATG7and this gene’s related pathways include Autophagy and antigen processing and presentation mediated by MHC Class I (Stelzer et al., 2016). Although the relationship between ATG7 and schizophrenia has yet to be studied, Genecards shows that this gene is significantly associated with neuronal abnormalities and mental illness, i.e., Neurodegeneration Caused by Brain Iron Accumulation, Huntington’s Disease, and Other Conditions.

GSK3A (Glycogen Synthase Kinase 3 Alpha) is a gene responsible for protein coding. Pancreatic cancer and hepatocellular carcinoma are two diseases linked to GSK3A. GSK3A is reported to be 80% lower in lymphocytes of patients with schizophrenia and is a regulatory enzyme of some neuronal proteins associated with schizophrenia abnormalities (Nadri et al., 2003). This discovery was confirmed by Stephen et al., who proposed GSK3A as a schizophrenia biomarker in blood identification (Glatt et al., 2005).

ACVR1 (Activin A Receptor Type 1) is a gene that codes for protein. Fibrodysplasia Ossificans Progressiva and Epicanthus are two diseases linked to ACVR1. This gene has been proved in genome-wide association research of schizophrenia by Lee et al. (2013) to be involved in rs1146031 to ACVR1 to mesoderm formation and activin binding potential pathways (*p* < 0.001, FDR = 0.032, 0.034). Class A comprising rhodopsin-like receptors includes CX3CR1 which is a Gi protein-coupled receptor (GPCR) with seven transmembrane domains (Imai et al., 1997). CX3CR1 (40 kDa) is made up of 355 amino acid residues that form an extracellular N-terminus, alternately arranged α-helical domains (TM1-TM7), intracellular (IL1-IL3) and extracellular (EL1-EL3) loops, and an intracellular C-terminus (Raucci et al., 2014). CX3CR1 levels were found downregulated in schizophrenia and may be associated with a depression-anxiety phenotype (Bergon et al., 2015; Chamera et al., 2021).

According to our KEGG analysis, candidate genes are primarily enriched in the NF-κB signaling pathway, which plays a critical role in the pathophysiology of schizophrenia. According to the study, PACER levels were significantly lower in schizophrenia patients than in healthy subjects. It has been demonstrated that CTCF induces the expression of this lncRNA. Therefore, the inhibitory NF-B complex is blocked by PACER, thereby increasing the expression of COX-2 (Krawczyk and Emerson, 2014). The pairwise correlations between the lncRNAs and genes revealed significant correlations between each pair, which further confirms their involvement in a specific signaling pathway, namely the NF-B pathway. A robust correlation was observed between NKILA/ADINR and NKILA / HNF1A-AS1, suggesting that these genes have a close functional connection (Safa et al., 2020).

The six most relevant drugs (Aflatoxin B1, Valproic Acid, Arsenic, Benzo(a)pyrene, epigallocatechin gallate and Nickel) were selected through the gene-drug interaction network. Valproic Acid (VPA), a branched short-chain fatty acid extracted from naturally occurring valeric acid, is a commonly used drug for bipolar disorder (McIntyre et al., 2020). VPA exerts its pharmacodynamic effects in a variety of ways: it acts on γ amino butyric acid (GABA) levels in the brain, blocks voltage-gated ion channels, and also acts as an HDAC inhibitor (Ghodke-Puranik et al., 2013). Tatiana et al. discovered that VPA prevented overactivity as well as latency inhibition and prepulse defects in Disc1-L100P mice, and that glia numbers were also increased in the subventricular zone in these mice, which VPA normalized (Lipina et al., 2012). Epigallocatechin gallate is an extract from green tea. Green tea, a centuries-old beverage, consists of antioxidant polyphenols, majorly epigallocatechin-3-gallate (EGCG), which inhibits nitric oxide synthase (NOS) and the production of cytokines (Ahmed et al., 2002; Singal et al., 2006). It also improves learning and memory in old rats and has antidepressant and anti-anxiety properties (Vignes et al., 2006; Kaur et al., 2008; Sattayasai et al., 2008). An eight-week, randomized, double-blind study on the effects of EGCG on schizophrenia and bipolar disorder discovered that EGCG could achieve some treatment effect on negative symptoms compared to placebo, but did not induce any notable effect on positive symptoms or inflammatory markers (Loftis et al., 2013), indicating that more research is needed on the efficacy of EGCG on positive symptoms of schizophrenia.

Limitations of this study: Although the diagnostic prediction model performed well in this study it was not further validated in combination with experiments; it could not be analyzed along with clinical information due to insufficient corresponding clinical correlation studies.

## 5. Conclusion

Our study systematically discovered 10 candidate hub genes (DPF2, ATG7, GSK3A, TFDP2, ACVR1, CX3CR1, AP4M1, DEPDC5, NR4A2, and IKBKB). A good diagnostic prediction model was established through comprehensive analysis in both the training (AUC 0.91, CI 0.95–0.86) and validation group (AUC 0.94, CI 1.00–0.85). Drugs that may be useful in the treatment of schizophrenia were also obtained (Valproic Acid, Epigallocatechin gallate).

## Data availability statement

The original contributions presented in the study are included in the article/Supplementary material, further inquiries can be directed to the corresponding author.

## Author contributions

YF and JS wrote the main manuscript text. All authors contributed to the article and approved the submitted version.

## Conflict of interest

The authors declare that the research was conducted in the absence of any commercial or financial relationships that could be construed as a potential conflict of interest.

## Publisher’s note

All claims expressed in this article are solely those of the authors and do not necessarily represent those of their affiliated organizations, or those of the publisher, the editors and the reviewers. Any product that may be evaluated in this article, or claim that may be made by its manufacturer, is not guaranteed or endorsed by the publisher.

## Footnotes

^1^https://www.ncbi.nlm.nih.gov/geo/

^2^https://www.kegg.jp/kegg/rest/keggapi.html

^3^http://genemania.org

^4^http://bioinfo.life.hust.edu.cn/ImmuCellAI/#!/

^5^https://www.networkanalyst.ca/
